# Chlorido[1-phenyl-3-(2,3,5,6-tetra­methyl­benz­yl)benzimidazol-2-yl­idene]silver(I)

**DOI:** 10.1107/S1600536812012998

**Published:** 2012-04-13

**Authors:** Mehmet Akkurt, Senem Akkoç, Yetkin Gök, Yılmaz Dağdemir, Muhammad Nawaz Tahir

**Affiliations:** a Department of Physics, Faculty of Sciences, Erciyes University, 38039 Kayseri, Turkey; b Department of Chemistry, Faculty of Sciences, Erciyes University, 38039 Kayseri, Turkey; c Department of Chemistry, Faculty of Arts and Sciences, Ínönü University, 44280 Malatya, Turkey; d Department of Physics, University of Sargodha, Sargodha, Pakistan

## Abstract

In the title compound, [AgCl(C_24_H_24_N_2_)], the terminal phenyl and tetra­methyl­benzene rings [which form a dihedral angle of 87.92 (14)°] make dihedral angles of 59.59 (11) and 83.19 (12)° with respect to the central benzimidazole ring system. The Ag—C and Ag—Cl single-bond lengths are 2.087 (3) and 2.3267 (9) Å. The C—Ag—Cl bond angle is 172.84 (7)°. C—H⋯π inter­actions contribute to the stabilization of the crystal structure. A very weak π–π stacking inter­action between adjacent tetra­methyl­benzene rings [centroid–centroid distance = 4.0610 (18) Å] is also observed.

## Related literature

For the synthesis, see: Yigit *et al.* (2012 ▶); Özdemir *et al.* (2010*c* ▶). For applications of silver *N*-heterocyclic carbene complexes in synthesis, catalysis, nanomaterials, and biology, see: Arduengo *et al.* (1993 ▶); Guerret *et al.* (1997 ▶); Patil *et al.* (2011 ▶); Özdemir *et al.* (2010*b* ▶); Liao *et al.* (2008 ▶). For related compounds, see: Patil *et al.* (2010 ▶); Zhou *et al.* (2008 ▶); Berding *et al.* (2009 ▶). For bond-length data, see: Özdemir *et al.* (2010*a* ▶); Allen *et al.* (1987 ▶).
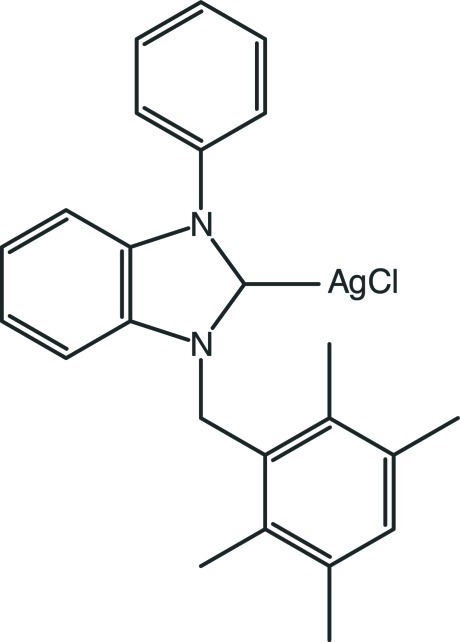


## Experimental

### 

#### Crystal data

[AgCl(C_24_H_24_N_2_)]
*M* *_r_* = 483.77
Monoclinic, 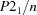

*a* = 9.1439 (2) Å
*b* = 18.7633 (4) Å
*c* = 13.2710 (3) Å
β = 109.899 (1)°
*V* = 2140.96 (8) Å^3^
*Z* = 4
Mo *K*α radiation
μ = 1.08 mm^−1^
*T* = 296 K
0.35 × 0.22 × 0.20 mm

#### Data collection

Bruker Kappa APEXII CCD diffractometer
Absorption correction: multi-scan (*SADABS*; Bruker, 2005 ▶) *T*_min_ = 0.752, *T*_max_ = 0.806
19846 measured reflections
5288 independent reflections
3354 reflections with *I* > 2σ(*I*)
*R*_int_ = 0.040

#### Refinement

*R*[*F* ^2^ > 2σ(*F* ^2^)] = 0.036
*wR*(*F* ^2^) = 0.080
*S* = 1.01
5288 reflections
257 parameters
H-atom parameters constrained
Δρ_max_ = 0.38 e Å^−3^
Δρ_min_ = −0.38 e Å^−3^

### 

Data collection: *APEX2* (Bruker, 2009 ▶); cell refinement: *SAINT* (Bruker, 2009 ▶); data reduction: *SAINT*; program(s) used to solve structure: *SHELXS97* (Sheldrick, 2008 ▶); program(s) used to refine structure: *SHELXL97* (Sheldrick, 2008 ▶); molecular graphics: *ORTEP-3 for Windows* (Farrugia, 1997 ▶) and *PLATON* (Spek, 2009 ▶); software used to prepare material for publication: *WinGX* (Farrugia, 1999 ▶) and *PLATON*.

## Supplementary Material

Crystal structure: contains datablock(s) global, I. DOI: 10.1107/S1600536812012998/sj5223sup1.cif

Structure factors: contains datablock(s) I. DOI: 10.1107/S1600536812012998/sj5223Isup2.hkl

Additional supplementary materials:  crystallographic information; 3D view; checkCIF report

## Figures and Tables

**Table 1:** Hydrogen-bond geometry (Å, °) *Cg*2 and *Cg*3 are the centroids of the C1–C6 benzene and C8–C13 phenyl rings, respectively.

*D*—H⋯*A*	*D*—H	H⋯*A*	*D*⋯*A*	*D*—H⋯*A*
C9—H9⋯*Cg*2^i^	0.93	2.69	3.507 (4)	147
C22—H22*A*⋯*Cg*3^ii^	0.96	2.80	3.525 (4)	133

Symmetry codes: (i) 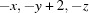; (ii) 
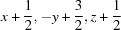
.

**Fig. 1.**
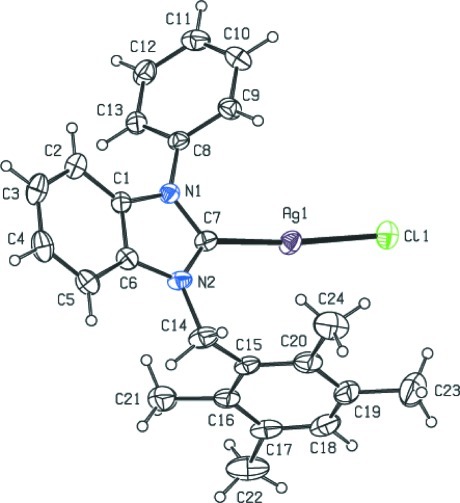
The title compound with the atom numbering scheme. Displacement ellipsoids for non-H atoms are drawn at the 30% probability level.

**Fig. 2.**
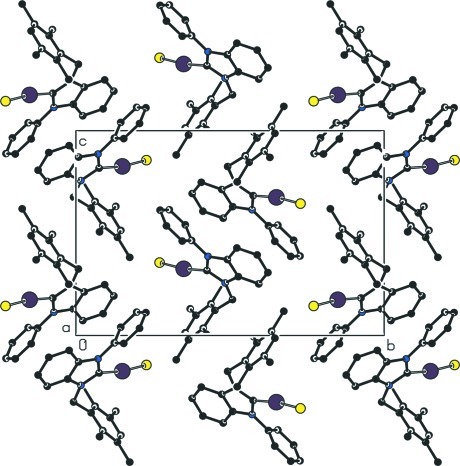
The packing of the title molecule in the unit cell, viewed along the a axis. H atoms are omitted for clarity.

## supplementary crystallographic information

### Comment

*N*-Heterocyclic carbene complexes (NHCs) have developed significantly in organometallic chemistry and homogenous catalysis since discovered, and have become extremely popular. Silver NHC complexes have particular interest because of their wide use as ligand transfer agents for the synthesis other metal-NHC complexes, catalysis, nanomaterials, and also biological activity as antimicrobial agents (Arduengo *et al.*, 1993; Guerret *et al.*, 1997; Patil *et al.*, 2011; Özdemir *et al.*, 2010*b*; Liao *et al.*, 2008; Patil *et al.*, 2010; Zhou *et al.*, 2008; Berding *et al.*, 2009).

In connection with our papers on the synthesis of the new complexes with *N*-heterocyclic carbene ligands, (Yigit *et al.*, 2012; Özdemir *et al.*, 2010*c*), we report here the crystal structure of the title compound, chlorido-[1-phenyl-3-(2,3,5,6-tetramethylbenzyl)benzimidazol-2-ylidene]silver (I).

In the title compound (I), (Fig. 1), the five- and six-membered rings (N1/N2/C1/C6/C7) and (C1–C6) of the benzimidazole groups are almost co-planar with maximum deviations of -0.012 (2) Å for N1 and 0.012 (3) Å for C6, respectively. The dihedral angle between them is 4.53 (16)°. The C8–C13 phenyl and C15–C20 benzene rings make dihedral angles of 59.59 (11)° and 83.19 (12)°, respectively, with respect to the mean plane of the central N1/N2/C1–C7 benzimidazole ring system, while they make a dihedral angle of 87.92 (14)° with each other. The Ag—C and Ag—Cl single bond lengths are 2.087 (3) Å and 2.3267 (9) Å. The C—Ag—Cl bond angle is 172.84 (7)°. The values of the geometrical parameters of (I) are in agreement with those reported for similar compounds (Allen *et al.*, 1987; Özdemir *et al.*, 2010*a*).

The crystal structure is stabilized by C—H···π interactions (Table 1) and weak π-π stacking interactions between adjacent (C15–C20: *Cg*4) benzene rings [*Cg*4···*Cg*4(1 - *x*, 2 - *y*, 1 - *z*) = 4.0610 (18) Å]. Fig. 2 shows the packing of (I) in the unit cell, viewed along the *a* axis.

### Experimental

For the originally reported synthesis, see: Yigit *et al.* (2012); Özdemir *et al.* (2010*c*). Single crystals of the title compound were obtained by recrystallization from dichloromethane/hexane at room temperature. (Yields: 0.281 g; 84%. M.p.: 524–525 K).

### Refinement

The H atoms were positioned geometrically with C—H = 0.93, C—H = 0.97 and C—H = 0.96 Å, for the aromatic, methylene and methyl H atoms, respectively and refined using a riding model with *U*_iso_(H) = *xU*_eq_(C), where *x* = 1.5 for the methyl H atoms and *x* = 1.2 for all other H atoms.

### Crystal data

**Table:**

[AgCl(C_24_H_24_N_2_)]	*F*(000) = 984
*M**_r_* = 483.77	*D*_x_ = 1.501 Mg m^−^^3^
Monoclinic, *P*2_1_/*n*	Mo *K*α radiation, λ = 0.71073 Å
Hall symbol: -P 2yn	Cell parameters from 3354 reflections
*a* = 9.1439 (2) Å	θ = 2.4–28.3°
*b* = 18.7633 (4) Å	µ = 1.08 mm^−^^1^
*c* = 13.2710 (3) Å	*T* = 296 K
β = 109.899 (1)°	Prism, white
*V* = 2140.96 (8) Å^3^	0.35 × 0.22 × 0.20 mm
*Z* = 4	

### Data collection

**Table:**

Bruker Kappa APEXII CCD diffractometer	5288 independent reflections
Radiation source: fine-focus sealed tube	3354 reflections with *I* > 2σ(*I*)
Graphite monochromator	*R*_int_ = 0.040
Detector resolution: 8.00 pixels mm^-1^	θ_max_ = 28.3°, θ_min_ = 2.4°
ω scans	*h* = −10→12
Absorption correction: multi-scan (*SADABS*; Bruker, 2005)	*k* = −25→16
*T*_min_ = 0.752, *T*_max_ = 0.806	*l* = −17→17
19846 measured reflections	

### Refinement

**Table:**

Refinement on *F*^2^	Primary atom site location: structure-invariant direct methods
Least-squares matrix: full	Secondary atom site location: difference Fourier map
*R*[*F*^2^ > 2σ(*F*^2^)] = 0.036	Hydrogen site location: inferred from neighbouring sites
*wR*(*F*^2^) = 0.080	H-atom parameters constrained
*S* = 1.01	*w* = 1/[σ^2^(*F*_o_^2^) + (0.0308*P*)^2^ + 0.160*P*] where *P* = (*F*_o_^2^ + 2*F*_c_^2^)/3
5288 reflections	(Δ/σ)_max_ = 0.001
257 parameters	Δρ_max_ = 0.38 e Å^−^^3^
0 restraints	Δρ_min_ = −0.38 e Å^−^^3^

### Special details

**Table:**

Experimental. *M*.p.: 524–525 K. n_(CN)_=1593.31 cm^-1. 1^H NMR (DMSO) δ: 2.11, 2.18 (s, 12H, NCH_2_C_6_H(C*H*_3_)_4_-2,3,5,6); 5.61 (s, 2H, NC*H*_2_C_6_H(CH_3_)_4_-2,3,5,6); 6.66–7.87 (m, 10H, Ar-*H*). ^13^C NMR (DMSO) d: 16.4, 20.8 (NCH_2_C_6_H(*C*H_3_)_4_-2,3,5,6); 55.2 (N*C*H_2_C_6_H(CH_3_)_4_-2,3,5,6); 112.5, 112.8, 124.9, 125.4, 126.8, 129.8, 130.4, 131.3, 132.7, 133.9, 134.6, 138.9 (Ar-*C*); the carbene carbon was not detected. Analysis calculated for C_24_H_24_N_2_AgCl: C 59.58, H 5.00, N 5.79%. Found: C 59.56, H 5.01, N 5.78%.
Geometry. Bond distances, angles *etc*. have been calculated using the rounded fractional coordinates. All su's are estimated from the variances of the (full) variance-covariance matrix. The cell e.s.d.'s are taken into account in the estimation of distances, angles and torsion angles
Refinement. Refinement on *F*^2^ for ALL reflections except those flagged by the user for potential systematic errors. Weighted *R*-factors *wR* and all goodnesses of fit *S* are based on *F*^2^, conventional *R*-factors *R* are based on *F*, with *F* set to zero for negative *F*^2^. The observed criterion of *F*^2^ > σ(*F*^2^) is used only for calculating -*R*-factor-obs *etc*. and is not relevant to the choice of reflections for refinement. *R*-factors based on *F*^2^ are statistically about twice as large as those based on *F*, and *R*-factors based on ALL data will be even larger.

### Fractional atomic coordinates and isotropic or equivalent isotropic displacement parameters (Å^2^)

**Table:**

	*x*	*y*	*z*	*U*_iso_*/*U*_eq_	
Ag1	0.25184 (3)	0.84953 (1)	0.17444 (2)	0.0476 (1)	
Cl1	0.43000 (9)	0.77142 (4)	0.14398 (7)	0.0572 (3)	
N1	−0.0611 (3)	0.92849 (11)	0.11403 (17)	0.0370 (7)	
N2	0.1099 (3)	0.98412 (11)	0.24355 (18)	0.0419 (8)	
C1	−0.1317 (3)	0.99141 (14)	0.1290 (2)	0.0383 (9)	
C2	−0.2743 (3)	1.02215 (16)	0.0728 (3)	0.0516 (11)	
C3	−0.3022 (4)	1.08812 (18)	0.1066 (3)	0.0633 (14)	
C4	−0.1939 (4)	1.12282 (17)	0.1930 (3)	0.0600 (13)	
C5	−0.0523 (4)	1.09315 (15)	0.2480 (2)	0.0490 (11)	
C6	−0.0233 (3)	1.02675 (14)	0.2127 (2)	0.0400 (9)	
C7	0.0885 (3)	0.92485 (14)	0.1829 (2)	0.0407 (9)	
C8	−0.1372 (3)	0.87529 (14)	0.0361 (2)	0.0365 (8)	
C9	−0.0741 (4)	0.85480 (15)	−0.0403 (2)	0.0458 (10)	
C10	−0.1480 (4)	0.80302 (16)	−0.1139 (2)	0.0552 (11)	
C11	−0.2846 (4)	0.77302 (16)	−0.1119 (2)	0.0552 (11)	
C12	−0.3471 (4)	0.79363 (16)	−0.0351 (3)	0.0536 (11)	
C13	−0.2733 (3)	0.84494 (14)	0.0388 (2)	0.0443 (10)	
C14	0.2555 (4)	1.00783 (17)	0.3248 (2)	0.0575 (11)	
C15	0.3702 (3)	0.95064 (15)	0.3774 (2)	0.0452 (10)	
C16	0.3525 (4)	0.91300 (16)	0.4642 (2)	0.0480 (10)	
C17	0.4668 (4)	0.86531 (16)	0.5207 (2)	0.0579 (11)	
C18	0.5916 (4)	0.85445 (17)	0.4869 (3)	0.0675 (12)	
C19	0.6107 (4)	0.8891 (2)	0.4013 (3)	0.0639 (11)	
C20	0.4998 (4)	0.93808 (17)	0.3455 (2)	0.0556 (11)	
C21	0.2094 (4)	0.9235 (2)	0.4940 (3)	0.0758 (14)	
C22	0.4607 (5)	0.8255 (2)	0.6185 (3)	0.0917 (18)	
C23	0.7508 (5)	0.8708 (3)	0.3695 (4)	0.115 (2)	
C24	0.5189 (5)	0.9767 (2)	0.2499 (3)	0.0853 (17)	
H2	−0.34700	0.99920	0.01520	0.0620*	
H3	−0.39630	1.11050	0.07090	0.0760*	
H4	−0.21850	1.16730	0.21380	0.0720*	
H5	0.02020	1.11610	0.30560	0.0590*	
H9	0.01720	0.87570	−0.04210	0.0550*	
H10	−0.10550	0.78830	−0.16490	0.0660*	
H11	−0.33500	0.73880	−0.16250	0.0660*	
H12	−0.43850	0.77290	−0.03330	0.0640*	
H13	−0.31510	0.85920	0.09040	0.0530*	
H14A	0.22980	1.03330	0.38010	0.0690*	
H14B	0.30560	1.04140	0.29150	0.0690*	
H18	0.66720	0.82180	0.52420	0.0810*	
H21A	0.22080	0.96590	0.53640	0.1140*	
H21B	0.19590	0.88320	0.53450	0.1140*	
H21C	0.12030	0.92820	0.43010	0.1140*	
H22A	0.37210	0.79440	0.59830	0.1370*	
H22B	0.45230	0.85900	0.67090	0.1370*	
H22C	0.55390	0.79790	0.64840	0.1370*	
H23A	0.71950	0.83940	0.30890	0.1730*	
H23B	0.82820	0.84780	0.42840	0.1730*	
H23C	0.79320	0.91370	0.35120	0.1730*	
H24A	0.59720	1.01290	0.27490	0.1280*	
H24B	0.42190	0.99840	0.20850	0.1280*	
H24C	0.54970	0.94330	0.20620	0.1280*	

### Atomic displacement parameters (Å^2^)

**Table:**

	*U*^11^	*U*^22^	*U*^33^	*U*^12^	*U*^13^	*U*^23^
Ag1	0.0405 (1)	0.0420 (2)	0.0554 (2)	0.0047 (1)	0.0098 (1)	−0.0002 (1)
Cl1	0.0532 (5)	0.0540 (5)	0.0682 (5)	0.0093 (4)	0.0255 (4)	0.0036 (4)
N1	0.0355 (12)	0.0364 (13)	0.0371 (12)	0.0020 (11)	0.0096 (11)	−0.0018 (10)
N2	0.0431 (14)	0.0362 (14)	0.0375 (13)	−0.0034 (11)	0.0021 (11)	−0.0040 (11)
C1	0.0378 (15)	0.0395 (17)	0.0397 (16)	0.0031 (14)	0.0158 (14)	0.0020 (13)
C2	0.0395 (17)	0.055 (2)	0.060 (2)	0.0036 (15)	0.0164 (16)	0.0027 (16)
C3	0.048 (2)	0.053 (2)	0.090 (3)	0.0156 (17)	0.025 (2)	0.0087 (19)
C4	0.071 (2)	0.0398 (18)	0.083 (3)	0.0081 (19)	0.044 (2)	−0.0009 (18)
C5	0.062 (2)	0.0382 (18)	0.0521 (19)	−0.0015 (16)	0.0265 (18)	−0.0025 (14)
C6	0.0484 (17)	0.0342 (16)	0.0389 (16)	0.0035 (14)	0.0169 (14)	0.0053 (12)
C7	0.0400 (16)	0.0394 (17)	0.0381 (15)	−0.0002 (14)	0.0074 (14)	0.0011 (13)
C8	0.0363 (15)	0.0365 (15)	0.0317 (14)	0.0037 (13)	0.0051 (13)	0.0024 (12)
C9	0.0493 (17)	0.0513 (19)	0.0390 (15)	−0.0054 (15)	0.0179 (14)	−0.0013 (14)
C10	0.074 (2)	0.054 (2)	0.0415 (18)	−0.0010 (18)	0.0246 (18)	−0.0060 (15)
C11	0.063 (2)	0.0477 (19)	0.0437 (18)	−0.0020 (17)	0.0036 (17)	−0.0072 (15)
C12	0.0391 (17)	0.053 (2)	0.066 (2)	−0.0026 (15)	0.0143 (17)	−0.0038 (17)
C13	0.0399 (16)	0.0464 (18)	0.0474 (17)	0.0000 (15)	0.0161 (14)	−0.0064 (14)
C14	0.057 (2)	0.0458 (19)	0.0532 (19)	−0.0074 (17)	−0.0028 (16)	−0.0059 (15)
C15	0.0407 (17)	0.0428 (18)	0.0400 (16)	−0.0072 (14)	−0.0018 (14)	−0.0065 (14)
C16	0.0478 (18)	0.0485 (19)	0.0402 (17)	−0.0133 (15)	0.0054 (15)	−0.0108 (14)
C17	0.066 (2)	0.049 (2)	0.0400 (17)	−0.0114 (17)	−0.0063 (17)	−0.0023 (15)
C18	0.057 (2)	0.056 (2)	0.064 (2)	0.0075 (18)	−0.0126 (19)	−0.0112 (18)
C19	0.0424 (19)	0.074 (2)	0.066 (2)	−0.0081 (19)	0.0065 (18)	−0.022 (2)
C20	0.056 (2)	0.056 (2)	0.0476 (18)	−0.0213 (17)	0.0082 (17)	−0.0109 (16)
C21	0.064 (2)	0.100 (3)	0.061 (2)	−0.014 (2)	0.018 (2)	−0.015 (2)
C22	0.123 (4)	0.074 (3)	0.052 (2)	−0.023 (3)	−0.004 (2)	0.0150 (19)
C23	0.057 (3)	0.153 (5)	0.131 (4)	0.002 (3)	0.026 (3)	−0.044 (4)
C24	0.092 (3)	0.098 (3)	0.068 (3)	−0.037 (3)	0.030 (2)	−0.003 (2)

### Geometric parameters (Å, º)

**Table:**

Ag1—Cl1	2.3267 (9)	C19—C20	1.382 (5)
Ag1—C7	2.087 (3)	C19—C23	1.518 (6)
N1—C1	1.392 (4)	C20—C24	1.522 (5)
N1—C7	1.364 (4)	C2—H2	0.9300
N1—C8	1.435 (3)	C3—H3	0.9300
N2—C6	1.397 (4)	C4—H4	0.9300
N2—C7	1.348 (3)	C5—H5	0.9300
N2—C14	1.468 (4)	C9—H9	0.9300
C1—C2	1.388 (4)	C10—H10	0.9300
C1—C6	1.381 (4)	C11—H11	0.9300
C2—C3	1.370 (5)	C12—H12	0.9300
C3—C4	1.395 (5)	C13—H13	0.9300
C4—C5	1.370 (5)	C14—H14A	0.9700
C5—C6	1.388 (4)	C14—H14B	0.9700
C8—C9	1.381 (4)	C18—H18	0.9300
C8—C13	1.380 (4)	C21—H21A	0.9600
C9—C10	1.380 (4)	C21—H21B	0.9600
C10—C11	1.379 (5)	C21—H21C	0.9600
C11—C12	1.383 (5)	C22—H22A	0.9600
C12—C13	1.376 (4)	C22—H22B	0.9600
C14—C15	1.497 (4)	C22—H22C	0.9600
C15—C16	1.407 (4)	C23—H23A	0.9600
C15—C20	1.407 (5)	C23—H23B	0.9600
C16—C17	1.386 (4)	C23—H23C	0.9600
C16—C21	1.503 (5)	C24—H24A	0.9600
C17—C18	1.376 (5)	C24—H24B	0.9600
C17—C22	1.515 (5)	C24—H24C	0.9600
C18—C19	1.371 (5)		
			
Cl1—Ag1—C7	172.84 (7)	C2—C3—H3	119.00
C1—N1—C7	110.8 (2)	C4—C3—H3	119.00
C1—N1—C8	123.9 (2)	C3—C4—H4	119.00
C7—N1—C8	125.3 (2)	C5—C4—H4	119.00
C6—N2—C7	111.3 (2)	C4—C5—H5	122.00
C6—N2—C14	121.6 (2)	C6—C5—H5	122.00
C7—N2—C14	126.9 (3)	C8—C9—H9	120.00
N1—C1—C2	132.2 (3)	C10—C9—H9	120.00
N1—C1—C6	106.3 (2)	C9—C10—H10	120.00
C2—C1—C6	121.2 (3)	C11—C10—H10	120.00
C1—C2—C3	116.4 (3)	C10—C11—H11	120.00
C2—C3—C4	122.1 (3)	C12—C11—H11	120.00
C3—C4—C5	121.8 (3)	C11—C12—H12	120.00
C4—C5—C6	116.1 (3)	C13—C12—H12	120.00
N2—C6—C1	106.0 (2)	C8—C13—H13	120.00
N2—C6—C5	131.5 (3)	C12—C13—H13	120.00
C1—C6—C5	122.4 (3)	N2—C14—H14A	108.00
Ag1—C7—N1	124.81 (18)	N2—C14—H14B	108.00
Ag1—C7—N2	129.1 (2)	C15—C14—H14A	108.00
N1—C7—N2	105.5 (2)	C15—C14—H14B	108.00
N1—C8—C9	120.1 (3)	H14A—C14—H14B	107.00
N1—C8—C13	119.2 (2)	C17—C18—H18	118.00
C9—C8—C13	120.7 (3)	C19—C18—H18	118.00
C8—C9—C10	119.3 (3)	C16—C21—H21A	109.00
C9—C10—C11	120.2 (3)	C16—C21—H21B	109.00
C10—C11—C12	120.3 (3)	C16—C21—H21C	109.00
C11—C12—C13	119.7 (3)	H21A—C21—H21B	110.00
C8—C13—C12	119.9 (3)	H21A—C21—H21C	109.00
N2—C14—C15	116.2 (2)	H21B—C21—H21C	110.00
C14—C15—C16	119.0 (3)	C17—C22—H22A	110.00
C14—C15—C20	120.5 (3)	C17—C22—H22B	109.00
C16—C15—C20	120.4 (3)	C17—C22—H22C	109.00
C15—C16—C17	119.4 (3)	H22A—C22—H22B	109.00
C15—C16—C21	120.2 (3)	H22A—C22—H22C	109.00
C17—C16—C21	120.4 (3)	H22B—C22—H22C	109.00
C16—C17—C18	118.4 (3)	C19—C23—H23A	109.00
C16—C17—C22	122.7 (3)	C19—C23—H23B	109.00
C18—C17—C22	118.9 (3)	C19—C23—H23C	109.00
C17—C18—C19	123.6 (3)	H23A—C23—H23B	109.00
C18—C19—C20	118.8 (3)	H23A—C23—H23C	110.00
C18—C19—C23	118.7 (4)	H23B—C23—H23C	109.00
C20—C19—C23	122.4 (4)	C20—C24—H24A	109.00
C15—C20—C19	119.3 (3)	C20—C24—H24B	109.00
C15—C20—C24	121.2 (3)	C20—C24—H24C	109.00
C19—C20—C24	119.5 (3)	H24A—C24—H24B	109.00
C1—C2—H2	122.00	H24A—C24—H24C	109.00
C3—C2—H2	122.00	H24B—C24—H24C	110.00
			
C7—N1—C1—C2	172.8 (3)	C4—C5—C6—C1	−1.7 (4)
C8—N1—C1—C2	−7.1 (5)	N1—C8—C9—C10	179.3 (2)
C7—N1—C1—C6	−2.1 (3)	C9—C8—C13—C12	0.1 (4)
C8—N1—C1—C6	178.0 (2)	C13—C8—C9—C10	−0.5 (4)
C1—N1—C7—Ag1	−169.48 (19)	N1—C8—C13—C12	−179.7 (3)
C8—N1—C7—Ag1	10.4 (4)	C8—C9—C10—C11	1.0 (4)
C1—N1—C7—N2	2.2 (3)	C9—C10—C11—C12	−1.1 (5)
C8—N1—C7—N2	−178.0 (2)	C10—C11—C12—C13	0.8 (5)
C7—N1—C8—C13	122.9 (3)	C11—C12—C13—C8	−0.3 (4)
C1—N1—C8—C9	123.0 (3)	N2—C14—C15—C16	−83.2 (3)
C7—N1—C8—C9	−56.9 (4)	N2—C14—C15—C20	101.2 (3)
C1—N1—C8—C13	−57.2 (4)	C14—C15—C16—C17	−173.2 (3)
C7—N2—C14—C15	−26.7 (4)	C14—C15—C16—C21	7.9 (4)
C6—N2—C7—N1	−1.4 (3)	C20—C15—C16—C17	2.4 (4)
C14—N2—C7—N1	−175.3 (3)	C20—C15—C16—C21	−176.5 (3)
C6—N2—C14—C15	160.1 (3)	C14—C15—C20—C19	174.6 (3)
C14—N2—C6—C1	174.4 (2)	C14—C15—C20—C24	−6.2 (4)
C7—N2—C6—C5	−176.4 (3)	C16—C15—C20—C19	−0.9 (4)
C14—N2—C6—C5	−2.2 (5)	C16—C15—C20—C24	178.3 (3)
C6—N2—C7—Ag1	169.7 (2)	C15—C16—C17—C18	−2.5 (4)
C14—N2—C7—Ag1	−4.1 (4)	C15—C16—C17—C22	176.8 (3)
C7—N2—C6—C1	0.2 (3)	C21—C16—C17—C18	176.4 (3)
N1—C1—C2—C3	−175.7 (3)	C21—C16—C17—C22	−4.3 (5)
N1—C1—C6—C5	178.1 (3)	C16—C17—C18—C19	1.1 (5)
C6—C1—C2—C3	−1.5 (5)	C22—C17—C18—C19	−178.2 (3)
C2—C1—C6—C5	2.5 (4)	C17—C18—C19—C20	0.4 (5)
C2—C1—C6—N2	−174.4 (3)	C17—C18—C19—C23	−178.0 (4)
N1—C1—C6—N2	1.1 (3)	C18—C19—C20—C15	−0.5 (5)
C1—C2—C3—C4	−0.2 (5)	C18—C19—C20—C24	−179.7 (3)
C2—C3—C4—C5	1.0 (6)	C23—C19—C20—C15	177.9 (3)
C3—C4—C5—C6	0.0 (5)	C23—C19—C20—C24	−1.3 (5)
C4—C5—C6—N2	174.4 (3)		

### Hydrogen-bond geometry (Å, º)

Cg2 and Cg3 are the centroids of the C1–C6 benzene and C8–C13 phenyl rings, respectively.

**Table:**

*D*—H···*A*	*D*—H	H···*A*	*D*···*A*	*D*—H···*A*
C9—H9···*Cg*2^i^	0.93	2.69	3.507 (4)	147
C22—H22*A*···*Cg*3^ii^	0.96	2.80	3.525 (4)	133

Symmetry codes: (i) −*x*, −*y*+2, −*z*; (ii) *x*+1/2, −*y*+3/2, *z*+1/2.
